# Whole-Genome Resequencing Reveals Genetic Diversity and Selective Sweep Signatures in Wuling Cattle

**DOI:** 10.3390/genes17091141

**Published:** 2026-09-18

**Authors:** Kunyou Xian, Yuee Gao, Zhiyi Su, Yating Wei, Kai Yang, Zexuan Hong, Xiaoqian Wu, Jicai Zhang, Halima Jafari, Chuzhao Lei, Ankui Wang

**Affiliations:** 1 Key Laboratory of Beef Cattle Genetics, Breeding and Reproduction Technology of Yunan Province, Yunan Academy of Grassland and Animal Science, Kunming 650000, China; porta1@163.com (K.X.); gye11722@126.com (Y.G.); yk123456ai@163.com (K.Y.); hongzexuan13@163.com (Z.H.); wxx2267464480@163.com (X.W.); ynzjc@126.com (J.Z.); 2 College of Animal Science and Technology, Northwest A&F University, Yangling 712100, China; 13213715269@163.com (Z.S.); weiyating315@163.com (Y.W.); halima.jafari@nwafu.edu.cn (H.J.)

**Keywords:** Wuling, Enshi, Sinan, Xiangxi, whole-genome resequencing, selective sweep analysis

## Abstract

**Background**: Wuling cattle are a native breed in southern China and primarily inhabit mountainous regions. They are distributed across three geographically distinct mountainous regions and were historically classified into three local populations: Enshi cattle (western Hubei), Sinan cattle (eastern Guizhou) and Xiangxi cattle (western Hunan). Here, we aim to elucidate the genetic relationships and describe the genetic characteristics of Wuling cattle across these regions. **Methods**: We performed whole-genome resequencing of 20 Sinan cattle, together with public data of 10 Enshi cattle, 20 Xiangxi cattle and 65 cattle from four reference continental groups. Reads were aligned to the ARS-UCD1.2 reference genome. Genetic differences between different cattle breeds were assessed through analyses of population structure and genetic diversity. Selective signals in Wuling cattle were identified using single-population selective analysis and local ancestry inference, whilst potential differences between different Wuling cattle subpopulations were pinpointed using cross-population selective analysis. **Results**: On the basis of whole-genome resequencing data, Enshi, Sinan and Xiangxi cattle share highly similar genetic backgrounds (with similar ancestral lineages comprising East Asian taurine and East Asian indicine) and negligible genetic differentiation (the pairwise *F_ST_* among Enshi cattle, Sinan cattle and Xiangxi cattle was approximately 0.01), supporting their classification as a single breed—Wuling cattle. Selective sweep analysis in Wuling cattle revealed multiple genomic regions under selection associated with key biological processes, including reproduction, fat deposition, feed efficiency, skeletal and muscle development, and immune response. Part of these regions is derived from East Asian indicine, related with *SYTL1*, *TMEM222*, *WDTC1* and *SLC9A1*. Furthermore, Wuling cattle are found in various regions, where geographical conditions vary. Distinct selective signals were also observed among regional groups of Wuling cattle, likely reflecting adaptation to their diverse local environments. **Conclusions**: This study provides a comprehensive genomic characterization of Wuling cattle, outlines the selection signals for Wuling cattle, whilst demonstrating that different geographical environments have generated unique selection pressures within the Wuling cattle population. Providing a genetic guidelines for the conservation and sustainable utilization of their genetic resources.

## 1. Introduction

Cattle are widely distributed across the globe and provide essential food, draught power and other products. Cattle are broadly classified into two subspecies: taurine (Bos taurus) and indicine (Bos indicus) cattle [1]. Both taurine and indicine cattle were generated from the domestication of aurochs (Bos primigenius) 8000–10,000 years ago [2]. Taurine cattle are primarily found in temperate and cold regions, where they are well adapted to colder environments [3], whereas indicine cattle are predominantly located in tropical and subtropical regions, where they exhibit strong tolerance to heat and drought [4]. Studies have shown that global cattle populations originate from five ancestral lineages: European taurine (EUT), Eurasian taurine (EUAT), East Asian taurine (EAT), East Asian indicine (EAI) and South Asian indicine (SAI) [5].

Wuling cattle, a native breed of southern China, carry mixed ancestry from Bos taurus and Bos indicus [5] and are known for their tolerance of coarse feed and excellent climbing ability [6]. Their natural habitat is characterized by complex mountainous landscapes, where they are primarily raised under free-range conditions. Historically, Wuling cattle were classified into three populations on the basis of their geographic distribution: Enshi cattle (Hubei Province), Sinan cattle (Guizhou Province), and Xiangxi cattle (Hunan Province), each supported by its own conservation programme. Owing to their distribution across Hunan, Hubei and Guizhou Provinces, these groups are traditionally considered distinct breeds. However, despite their collective designation as Wuling cattle, the absence of comprehensive publicly available genomic data has impeded a clear understanding of their genetic relationships and hindered the development of effective conservation and breeding strategies.

Here, we performed whole-genome resequencing on 20 Sinan cattle, together with 95 public resequencing data (10 Enshi cattle, 20 Xiangxi cattle, 10 Hereford cattle, 10 Angus cattle, 15 Hanwoo, 20 Weizhou cattle and 10 Thawalam) to systematically characterize the population structure, genetic diversity and selective sweep signatures of Wuling cattle. The Enshi cattle and Xiangxi cattle have been analyzed as cattle breeds in southwest China, but there is a lack of specific understanding of the Wuling cattle breed itself. In this study, we assessed the genetic composition of Enshi cattle, Sinan cattle and Xiangxi cattle using global ancestor inference (by ADMIXTURE) and various genetic statistics. We also identified selective signals in Wuling cattle and among different Wuling cattle subgroups using methods based on site frequency spectrum (CLR) and extended haplotype homozygosity (iHS and XP-EHH). This study provides comprehensive genomic evidence to clarify the taxonomic status of Wuling cattle and explores the insights into the effects of different geographical environments on the internal characteristics of Wuling cattle, providing valuable genetic resources for their conservation and breeding utilization.

## 2. Materials and Methods

### 2.1. Samples and Sequencing

In this study, a total of 50 Wuling cattle were included for genomic analysis, comprising 20 newly sequenced Sinan cattle (the average sequencing depth is 17.4X) and 30 publicly available individuals (10 Enshi cattle and 20 Xiangxi cattle) (see Supplementary Material File S1 Table S1). Specifically, blood samples from 20 Sinan cattle were collected, and genomic DNA was extracted via the standard phenol–chloroform method. Whole-genome sequencing (WGS) was subsequently performed via the Illumina NovaSeq platform (Illumina, Inc., San Diego, CA, USA). Additionally, to investigate potential ancestral components and better characterize genetic diversity, 65 samples from four representative continental cattle groups were included as reference populations, following Chen et al. [5]. These groups included European taurine (10 Angus cattle and 10 Hereford cattle), East Asian taurine (15 Hanwoo), South Asian indicine (10 Thawalam), and East Asian indicine (20 Weizhou cattle) (see Supplementary Material File S1 Table S1). In total, 115 samples were used in this study.

### 2.2. Read Mapping and Single-Nucleotide Polymorphism (SNP) Calling

The raw reads were trimmed via Trimmomatic (v0.38) with the following parameters: LEADING:20, TRAILING:20, SLIDINGWINDOW:3:15, AVGQUAL:20, MINLEN:35, and TOPHRED33. The trimmed reads were then aligned to the ARS-UCD1.2 reference genome via BWA-MEM (v0.7.19), followed by sorting and duplicate removal via SortSam (v3.8) and MarkDuplicates (v3.8). Base quality score recalibration was performed via the GATK BaseRecalibrator (v3.8). The average sequencing depth for all the samples was calculated via Qualimap (v2.3) [7]. To identify SNPs across all samples, we applied GATK (v3.8) tools: HaplotypeCaller, GenotypeGVCFs and SelectVariants. Raw SNP calls were filtered with GATK VariantFiltration (v3.8) on the basis of the recommended hard filter criteria: (1) DP < 1/3× or DP > 3× (where × represents the sum of the average sequencing depths across all samples), (2) QD < 2.0, (3) FS > 60.0, (4) MQ < 40.0, (5) MQRankSum < −12.5, (6) ReadPosRankSum < −8.0, and (7) SOR > 3.0 [8]. We then used GATK4 (v4.0) SelectVariants and CatVariants to identify and merge all the biallelic SNPs across the samples. Missing genotypes were imputed and phased via BEAGLE v4.0 with default parameters. Linkage disequilibrium (LD) pruning was performed in PLINK with the options –maf 0.05 and –indep-pairwise 50 10 0.1 [9]. Finally, SNP annotation was conducted via ANNOVAR (v2025Mar02) with databases built on the ARS-UCD1.2 reference genome.

### 2.3. Global Ancestry Inference and Genetic Diversity Calculation

We performed global ancestry inference and principal component analysis (PCA) on the trimmed SNP dataset. Global ancestry inference was conducted with ADMIXTURE [10], and PCA was performed via the Smartpca module within EIGENSOFT (v4.2) [11] to investigate population structure. Pairwise genetic distances between SNPs were calculated with PLINK (v1.90) [12], and the resulting distance matrix was used in MEGA (v11.0) to construct a neighbor-joining (NJ) tree reflecting genetic relationships among the samples. Nucleotide diversity (PI) and the fixation index (*F_ST_*) across populations were computed via VCFtools (v0.1.16) [13]. LD decay between SNPs was estimated via PopLDdecay (v3.43). Runs of homozygosity (ROHs) were identified with PLINK’s (v1.90) –homozyg option via the following parameters: (1) –homozyg-window-snp 50, (2) –homozyg-snp 100, (3) –homozyg-kb 100, (4) –homozyg-gap 1000, (5) –homozyg-window-threshold 0.05, (6) –homozyg-window-het 1 [14]. ROH segments represent long contiguous homozygous regions inherited from shared parental haplotypes and can indicate recent inbreeding history. The identified ROH segments were categorized into three length classes (short, medium, and long) via a Gaussian mixture model. The *F_ROH_* inbreeding coefficient was calculated as the total length of medium and long ROH segments divided by the total genomic length [15].

### 2.4. Local Ancestry Inference

Local ancestry inference for the Wuling cattle was performed on the phased SNP dataset (phased by BEAGLE (v4.0) with default parameters) via Loter (v1.0.1) [16]. The output from Loter was processed via custom Python (v3.11) scripts to link adjacent loci inferred to have the same ancestral origin and calculate the proportion of ancestral fragments across all individuals. Genomic regions with fragment lengths greater than 100 bp, in which a single ancestry proportion exceeded 75%, were defined as ancestry-specific fragment regions [17].

### 2.5. Selective Sweep Test

Using the phased SNP dataset (phased by BEAGLE (v4.0) with default parameters), we performed selective sweep scans via three different statistics: the integrated haplotype score (iHS), the composite likelihood ratio (CLR) and cross-population extended haplotype homozygosity (XP-EHH). Selscan (v2.0) [18] was used to compute iHS and XP-EHH scores genome-wide. The raw iHS and XP-EHH outputs were normalized via the norm utility in Selscan (v2.0). Within 50 kb sliding windows with 20 kb steps, we calculated the fraction of extreme iHS scores and the maximum XP-EHH value for each window, reflecting local haplotype homozygosity and cross-population haplotype differences. SweepFinder2 (v3) [19] was applied to compute the CLR. CLR scores were also summarized in 50 kb windows with a 20 kb step, and the maximum CLR value per window was used as the measure of the local sweep signal. The top 1% of results from iHS and CLR were identified as candidate regions, while the candidate regions identified by both iHS and CLR were designated as the final selective regions. To capture interpopulation differences in selection, XP-EHH was used to compare the target population with reference populations, and windows with the top 5% of XP-EHH values were identified as regions with significant selective pressure differences. If a region identified by iHS and CLR analysis is located within this region, the relevant region is further identified as a selective region where selection differs across populations [20].

## 3. Results

### 3.1. Resequencing, Read Alignment and Detection of SNPs

We performed joint genotyping on whole-genome resequencing data from 50 Wuling cattle and 65 reference individuals via the ARS-UCD1.2 reference genome. The average sequencing depth across all samples was 12.63× (see Supplementary Material File S1 Table S1). After stringent filtering, 40.05 million biallelic variants were retained from the 115 samples. Annotation with ANNOVAR revealed that the majority of SNPs were located in intergenic regions (54.96%) and intronic regions (41.11%), with smaller proportions in upstream/downstream regions of open reading frames (1.66%) and exonic regions (0.78%) (see Supplementary Material File S1 Table S2). Among the exonic SNPs, 116,706 were nonsynonymous mutations, and 184,957 were synonymous mutations.

### 3.2. Population Structure and Genetic Diversity

**Figure 1:**
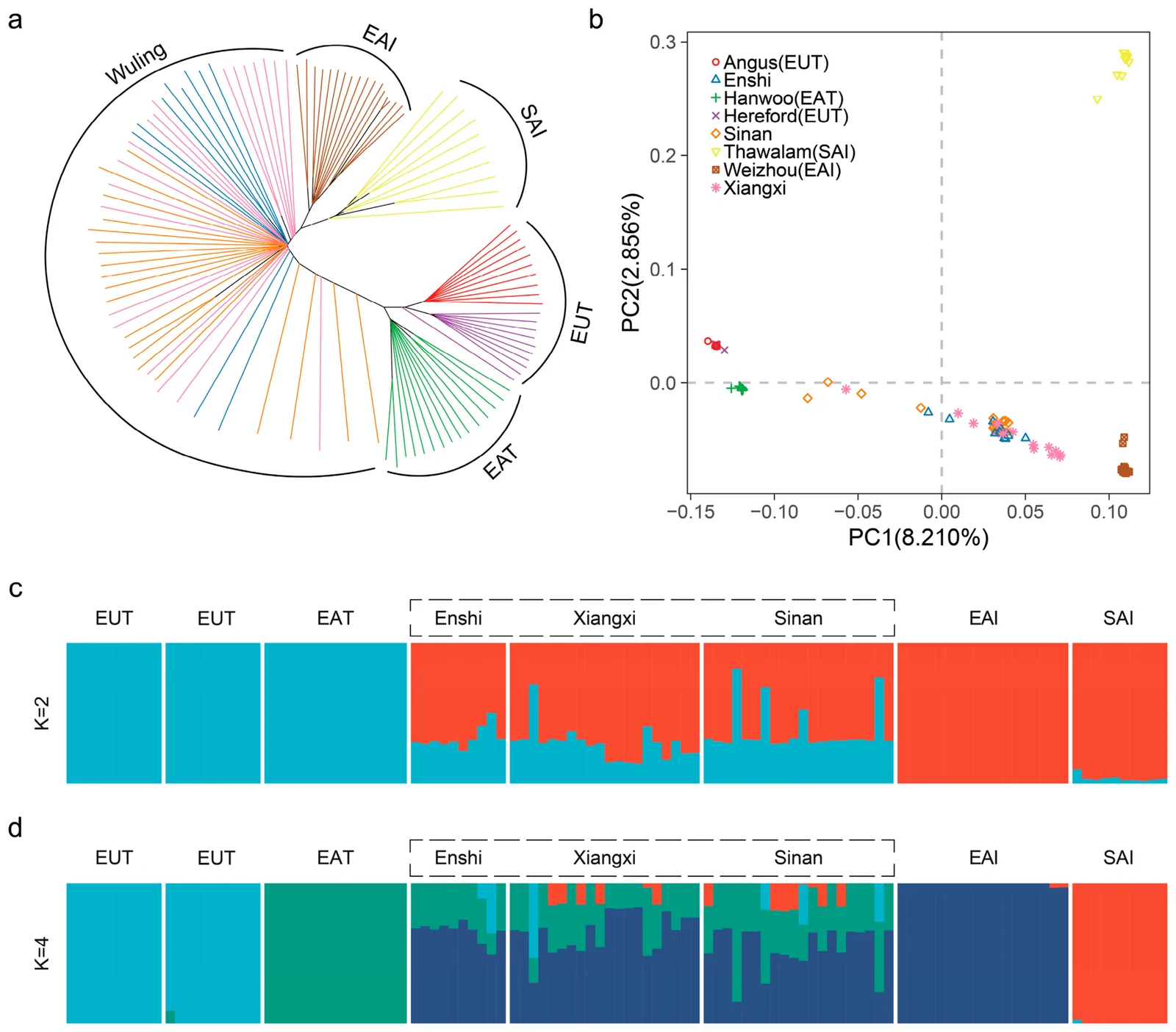
Population structure and relationships of Wuling cattle in comparison to several possible ancestral breeds. (**a**) Neighbor-joining tree depicting the relationships between Wuling cattle and potential ancestral breeds. The colors representing different breeds are consistent with those in (**b**). (**b**) PCA showing the genetic differentiation among Wuling cattle and reference breeds. (**c**,**d**) Model-based clustering of cattle breeds via ADMIXTURE for K = 2 and K = 4. Breeds are color-coded according to their geographic region and labeled with breed names.

Principal component analysis (PCA) and phylogenetic trees reproduced the well-established geographic differentiation among global cattle populations: European taurine (EUT), East Asian taurine (EAT), South Asian indicine (SAI), and East Asian indicine (EAI). The Enshi, Sinan and Xiangxi cattle did not form distinct clusters, indicating that their genetic compositions are highly similar (Figure 1a,b). ADMIXTURE analyses were performed for K values ranging from 2 to 8, where K represents the assumed number of ancestral populations. The lowest cross-validation (CV) errors were observed at K = 2 (CV = 0.22726) and K = 4 (CV = 0.23176). At K = 2, the samples were separated into the indicine and taurine groups. At K = 4, they were further subdivided into European taurine, East Asian taurine, East Asian indicine and South Asian indicine. The ADMIXTURE results confirmed that Enshi, Sinan and Xiangxi cattle share a similar ancestral composition, with major contributions from the EAT and EAI (Figure 1c,d).

**Figure 2:**
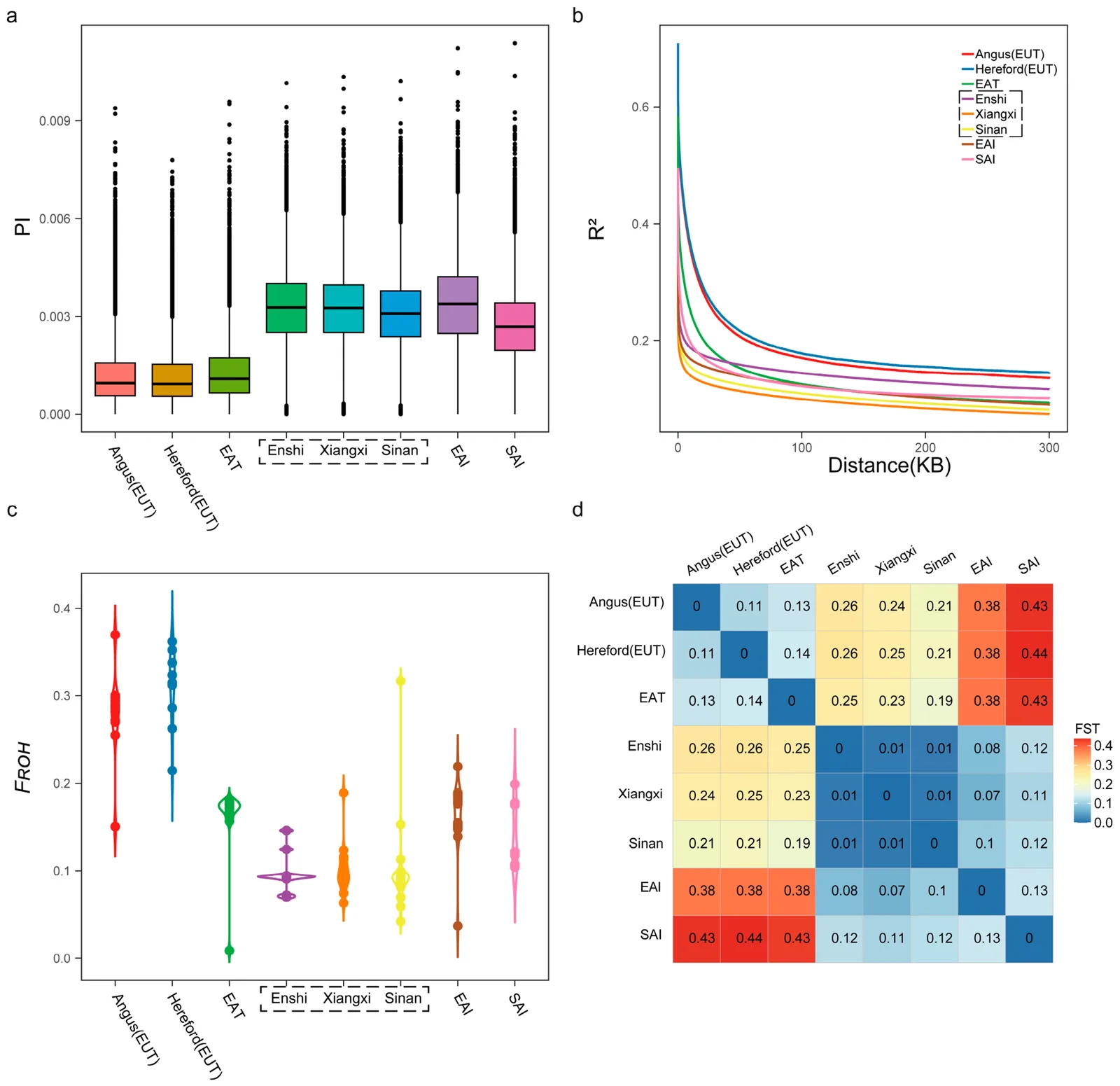
Genetic diversity of Enshi cattle, Sinan cattle and Xiangxi cattle in comparison to several possible ancestral breeds. (**a**) Nucleotide diversity (PI) for each group. (**b**) Linkage disequilibrium decay patterns (average R^2^ at different distances) for each group. (**c**) Genomic inbreeding coefficient (*F_ROH_*) for each group. (**d**) Average genome-wide *F_ST_* values between different groups.

To further investigate the genetic diversity of Enshi, Sinan, and Xiangxi cattle, we calculated PI, LD decay, the genomic inbreeding coefficient on the basis of runs of homozygosity (*F_ROH_*), and pairwise *F_ST_* between breeds (Figure 2a–d). The PI values were similar and relatively high across the three groups (Mean of PI in Enshi cattle: 0.00326398; Mean of PI in Sinan cattle: 0.00308982; Mean of PI in Xiangxi cattle: 0.00323943). LD decay patterns were largely comparable among Wuling cattle and most reference populations, whereas Angus and Hereford showed relatively slower LD decay. *F_ROH_*, an indicator of recent inbreeding, was low and comparable among the three groups (Mean of *F_ROH_* in Enshi cattle: 0.09697; Mean of *F_ROH_* in Sinan cattle: 0.100318; Mean of *F_ROH_* in Xiangxi cattle: 0.100098), suggesting limited inbreeding and similar breeding or management practices. *F_ST_* quantifies the degree of genomic differentiation between populations. In our study, the pairwise *F_ST_* among Enshi cattle, Sinan cattle and Xiangxi cattle was approximately 0.01, indicating extremely low population differentiation. This pattern confirms a shared genetic background among the three groups, with limited divergence despite their geographic separation.

Collectively, the genetic structure and diversity analyses provide strong evidence that Enshi cattle, Sinan cattle, and Xiangxi cattle share highly similar population structures and genetic compositions, with insufficient differentiation to support their classification as distinct breeds.

### 3.3. Genome-Wide Selective Sweep Signals in Wuling Cattle

**Figure 3:**
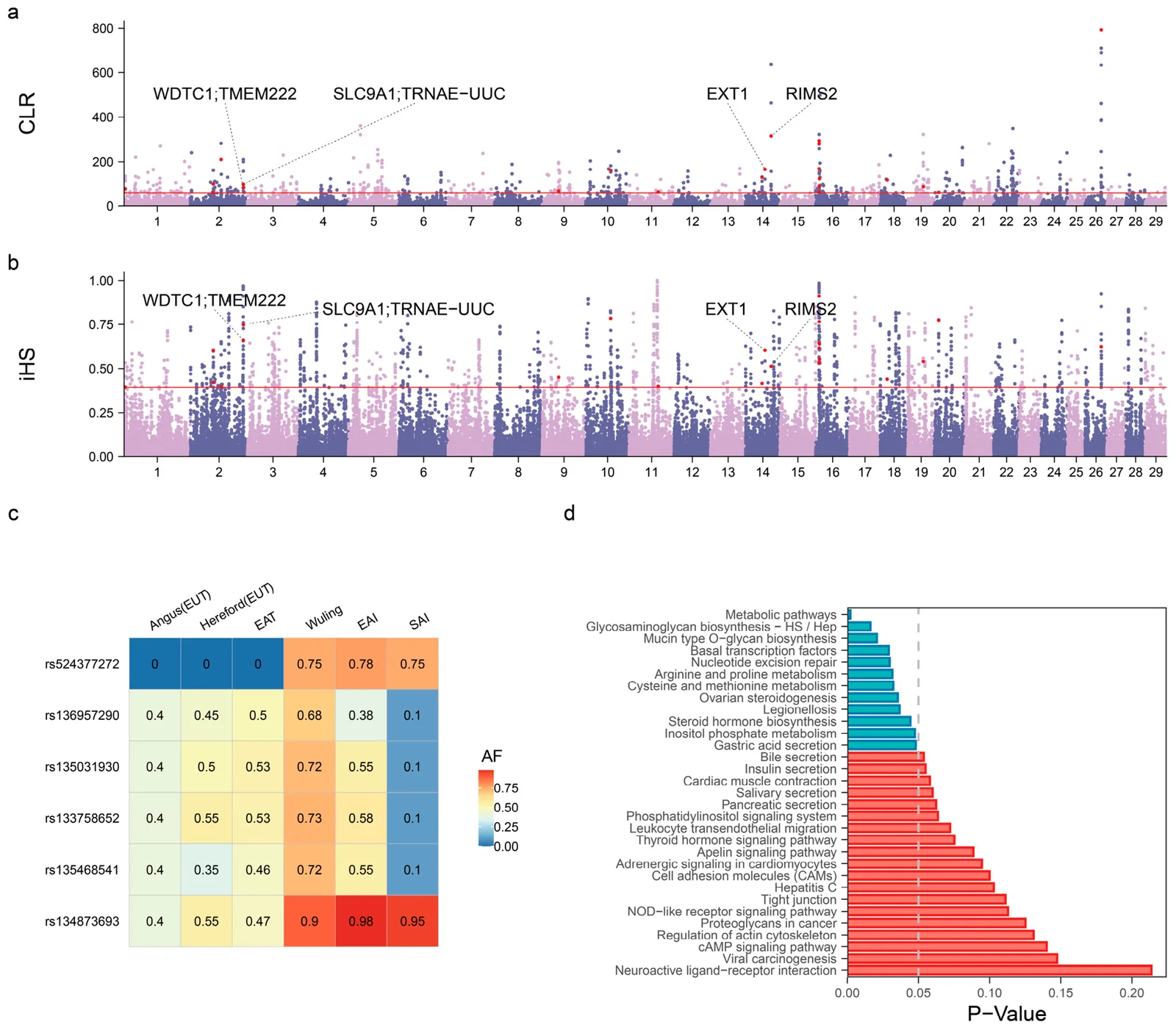
Genome-wide selective sweep analysis in Wuling cattle and functional annotation of candidate genes. (**a**) Manhattan plot displaying CLR values (*y*-axis) in 50 kb windows with 20 kb steps across all autosomes. Candidate regions are above the red line, selective regions are highlighted in red, and related genes are labeled. (**b**) Manhattan plot showing the extreme fraction of iHS (*y*-axis) in 50 kb windows with 20 kb steps across all autosomes. Candidate regions are above red line, selective regions are highlighted in red, related genes are labeled. (**c**) Allele frequencies of nonsynonymous mutations in different populations. (**d**) KEGG pathway enrichment analysis of candidate genes within selective regions. Pathways are ranked by *p* value (*x*-axis), with the dashed line indicating the nominal significance threshold (*p* = 0.05). Significant signaling pathways are colored blue.

Wuling cattle harbor ancestry from both EAT and EAI lineages, representing a unique genetic resource in southern China. To further characterize the genetic features of Wuling cattle, we combined samples from Enshi cattle, Sinan cattle and Xiangxi cattle into a single representative Wuling cattle dataset and conducted a selective sweep analysis. Genome-wide biallelic SNPs were evaluated via iHS and CLR. The results were summarized in 50 kb windows with a 20 kb step size, and the top 1% of the highest scoring windows were designated candidate regions. Candidate regions identified by both iHS and CLR were considered selective regions (Figure 3a,b).

In total, we detected 35 selective regions spanning approximately 1.84 Mb, containing 47 nonsynonymous variants (see Supplementary Material File S1 Tables S3 and S4) overlapping 29 genes. These genes are associated with several important traits, including reproduction (*AMD1* [21], *CYP19A1* [22]), feed utilization (*SLC9A1* [23]), skeletal development (*EXT1* [24], *RIMS2* [25]), muscle development (*KAT7* [26]), fat deposition (*WDTC1* [27], *TMEM222* [28]) and the immune response (*SYNJ1* [29]). Among the 47 nonsynonymous variants identified, six were further highlighted for their pronounced allele-frequency divergence in Wuling cattle: rs524377272 (Chr2: 125,940,682), located within *SYTL1*, and rs136957290 (Chr19: 36,796,203), rs135031930 (Chr19: 36,796,229), rs133758652 (Chr19: 36,801,337), rs135468541 (Chr19: 36,805,039) and rs134873693 (Chr19: 36,805,255), clustered within *TAC4* (Figure 3c). SYTL1 is implicated in vesicular trafficking and membrane docking [30], whereas TAC4 is closely associated with the regulation of immune and reproductive functions [31]. The relatively elevated allele frequencies of these variants observed in Wuling cattle suggest that they may contribute to breed-specific genetic differentiation. KEGG pathway enrichment analysis of the candidate genes revealed 35 associated pathways (Figure 3d). Pathways that reached nominal significance (*p* < 0.05) included glycosaminoglycan biosynthesis–heparan sulfate/heparin, steroid hormone biosynthesis, ovarian steroidogenesis, and inositol phosphate metabolism, which is consistent with the roles of candidate genes in skeletal development and reproductive regulation.

**Figure 4:**
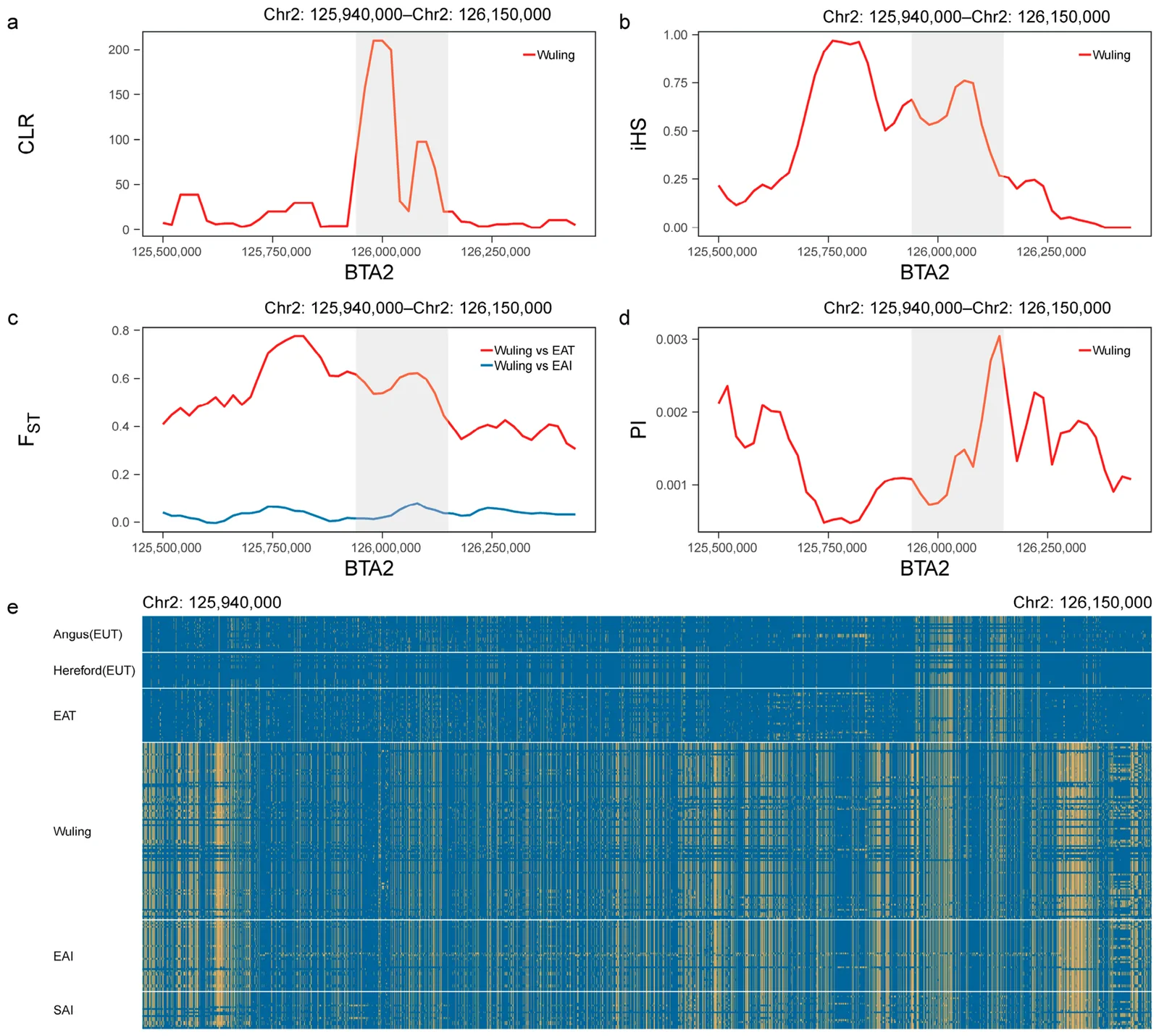
Examples of selective regions with excess indicine ancestry. (**a**) Line plot displaying CLR values (*y*-axis) for Wuling cattle across a selective region on BTA2. (**b**) Line plot showing the extreme fraction of iHS values (*y*-axis) for Wuling cattle on BTA2. (**c**) Line plot showing the *F_ST_* values (*y*-axis) between Wuling and Hanwoo and Weizhou cattle on BTA2. (**d**) Line plot displaying nucleotide diversity (PI) values (*y*-axis) for Wuling cattle on BTA2. (**e**) Haplotype patterns for the example region on BTA2 across six cattle breeds. Reference alleles are indicated in blue, and alternative alleles are indicated in yellow.

To further elucidate the respective contributions of EAT and EAI ancestry to the selective regions identified in Wuling cattle, we performed local ancestry inference (LAI) via Loter. Hanwoo (EAT) and Weizhou cattle (EAI) were used as reference panels to infer the ancestral origins of the genomic segments across the Wuling cattle genome. After excluding segments shorter than 100 bp, the Wuling cattle genome was partitioned into 1,451,095 independent segments, including 58,738 East Asian indicine-derived segments (with EAI ancestry ≥ 75%) and 512 East Asian taurine-derived segments (with EAT ancestry ≥ 75%). Notably, EAI-derived segments in Wuling cattle overlapped with selective regions involving eight genes (see Supplementary Material File S1 Table S5). These overlapping segments were predominantly concentrated in a 210 kb region on chromosome 2 (Chr2: 125,940,000–126,150,000), encompassing *SYTL1*, *TMEM222*, *WDTC1,* and *SLC9A1*, genes associated with fat deposition and feed utilization (Figure 4a–e). Notably, rs524377272 was also located within this region. Within this selective sweep, the top iHS signals mapped to two intronic variants in *SLC9A1*, located at Chr2: 126,101,646 (rs109046361) and Chr2: 126,114,246 (rs109807379). *SLC9A1* encodes Na^+^/H^+^ exchanger 1 (NHE1), a ubiquitously expressed major Na^+^/H^+^ antiporter at the mammalian plasma membrane, the expression of which has been reported to differ between dairy cows exhibiting distinct feed efficiency phenotypes [23].

Collectively, these results indicate detectable contributions from both EAT and EAI ancestries to the Wuling cattle genome. Moreover, only 4.08% of the genome was assigned to EAT- or EAI-derived segments. This finding highlights the distinctive genetic architecture of Wuling cattle and suggests a complex evolutionary history.

### 3.4. Genome-Wide Selective Sweep Signals in Subgroups of Wuling Cattle

Wuling cattle were historically classified into three separate groups: Enshi cattle, Sinan cattle and Xiangxi cattle. However, our results demonstrate that these three groups do not exhibit pronounced differences in genetic composition and should therefore be considered a single breed, Wuling cattle. While geographic variation may have imposed distinct local selective pressures [32], these differences do not warrant recognition as separate breeds. Nevertheless, they constitute important components of the Wuling cattle genetic resource.

**Figure 5:**
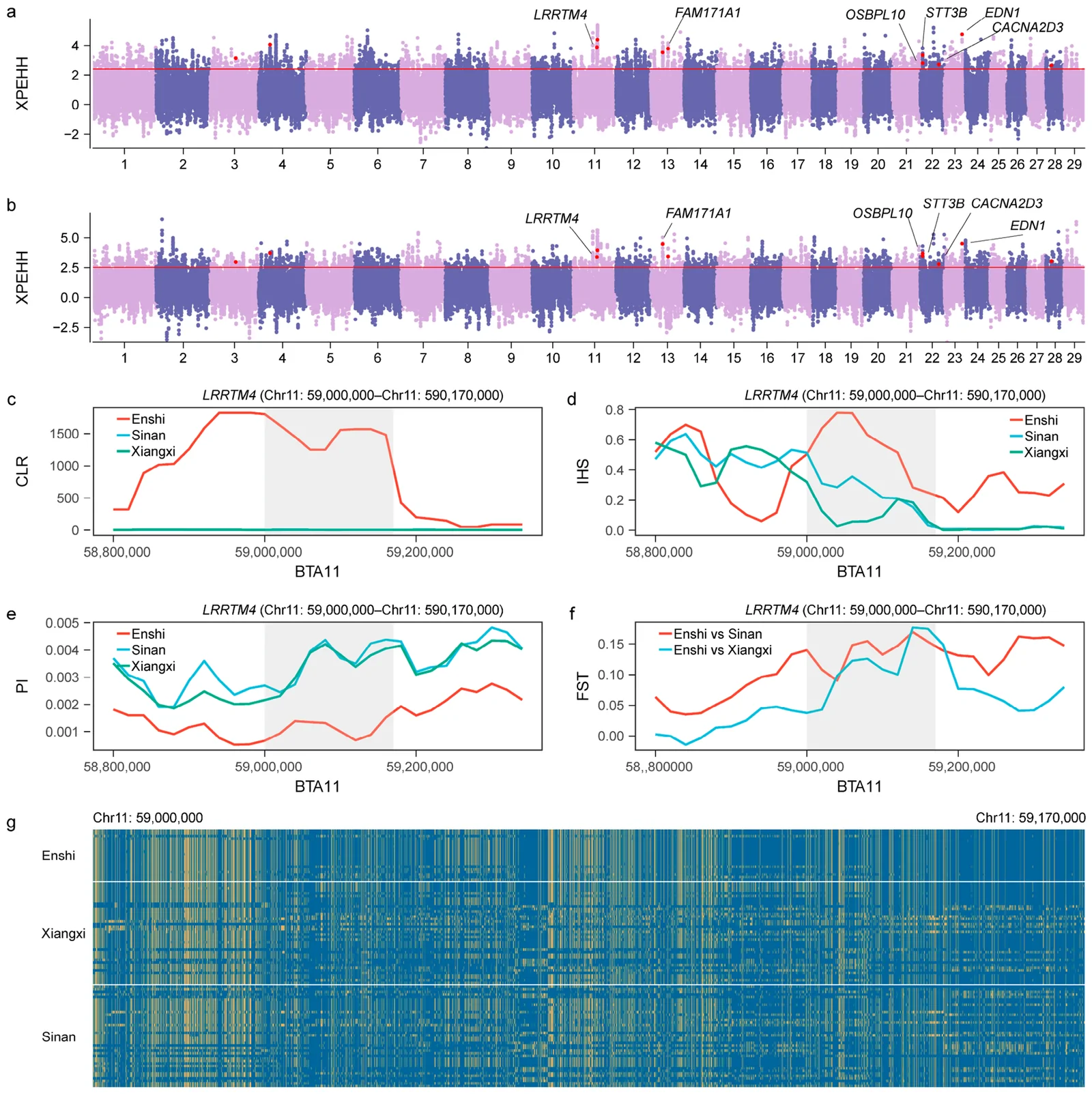
Example of selective regions. (**a**) Manhattan plot showing XP-EHH values (between Enshi cattle and Sinan cattle) in 50 kb windows with 20 kb steps across all autosomes. Candidate regions are above red line, selective regions are highlighted in red, related genes are labeled. (**b**) Manhattan plot showing XP-EHH values (between Enshi cattle and Xiangxi cattle) in 50 kb windows with 20 kb steps across all autosomes. Candidate regions are above red line, selective regions are highlighted in red, related genes are labeled. (**c**) CLR value at the selective region within the *LRRTM4* genes. (**d**) iHS value at the selective region within the *LRRTM4* genes. (**e**) Nucleotide diversity (PI) value at the selective region within the *LRRTM4* genes. (**f**) *F_ST_* value between Enshi cattle, Xiangxi cattle and Sinan cattle at the selective region within the *LRRTM4* genes. (**g**) Haplotype patterns for the selective region within *LRRTM4* at Enshi cattle, Xiangxi cattle and Sinan cattle. Reference alleles are indicated in blue, and alternative alleles are indicated in yellow.

To detect group-specific signatures, we applied the same selective sweep scan framework described above, using iHS and CLR to identify selective regions in each of the three constituent groups (see Supplementary Material File S1, Tables S6–S8). XP-EHH was used to assess differences in selective pressure among the different groups (Figure 5a,b).

In Enshi cattle, we identified 11 group-specific selective regions (see Supplementary Material File S1 Table S9), totaling approximately 990 kb and encompassing 18 genes associated with traits such as fat deposition (*OSBPL10* [33], *CACNA2D3* [34]), muscle development (*STT3B* [35]) and reproduction (*EDN1* [36], *LRRTM4* [37]). In Xiangxi cattle, we identified 3 group-specific selective regions (see Supplementary Material File S1 Table S10), totaling 150 kb and containing four genes, *DIO1*, *HSPB11*, *NCAM2* and *RALGAPA2*, which are implicated in fat deposition [38,39], reproduction [40], and the heat-stress response [41]. These regions exhibit selective signals within their respective groups and show substantially stronger selective signatures than the other two groups do, indicating heterogeneous selective pressures among the three Wuling cattle groups.

Notably, Enshi cattle harbor a long Enshi-specific selective segment within an intronic region of *LRRTM4* (Chr11: 59,000,000–59,170,000). This region displays a strong selective signature in Enshi cattle and shows clear genetic differentiation from Sinan and Xiangxi cattle (Figure 5c–g). The SNP with the highest iHS value was located at Chr11: 59,076,072 (rs453900543), within an intronic region of *LRRTM4*, which encodes a leucine-rich repeat transmembrane synaptic adhesion molecule. *LRRTM4* has been proposed as a candidate gene for high reproductive performance in goats on the basis of genome-wide selective signature analyses [37]. *LRRTM4* is also expressed in retinal rod bipolar cells, where it mediates inhibitory feedback synapses essential for dim-light vision [42]. Based on the results of the selective sweep analysis and existing relevant reports, we hypothesise that the mutations in *LRRTM4* may contribute to both increased fertility and low-light visual adaptation in Enshi cattle grazing in mountain environments, and this could be further validated in the future through experiments or transcriptomic analysis.

## 4. Discussion

China harbors more than fifty recognized indigenous cattle breeds, most of which were delimited during the twentieth century on the basis of morphology, production use and, above all, the administrative region in which they were reared [43]. Wuling cattle were historically classified into three local groups on the basis of their geographic rearing locations (Enshi, Sinan and Xiangxi) and were previously regarded as separate native breeds. However, our results demonstrate that these groups exhibit no pronounced differences in genetic composition and should instead be considered a single breed, Wuling cattle. Although geographic variation may have imposed distinct local selective pressures, these differences are insufficient to warrant recognition as separate breeds. Nonetheless, they represent valuable components of the overall Wuling cattle genetic resource.

Genome-wide analyses have revealed that Chinese indigenous cattle have diverse genetic backgrounds and ancestral compositions [43]. With respect to the population structure of Wuling cattle, we found that the Enshi cattle, Sinan cattle and Xiangxi cattle all carry mixed ancestry from the EAI and EAT lineages. Both the PCA and the NJ tree revealed that the three populations clustered together, reflecting highly similar population structures. Further genetic diversity analyses revealed reduced nucleotide diversity in breeds such as Angus, Hereford and Hanwoo, consistent with their histories of intensive artificial selection and pure-line breeding. In contrast, the three Wuling groups presented relatively high PI values, comparable to those of the Thawalam and Weizhou cattle, suggesting reduced artificial selection and the retention of substantial genetic variation. LD decay patterns and *F_ROH_* further support this conclusion. Moreover, pairwise *F_ST_* analyses revealed extremely low differentiation among Enshi cattle, Sinan cattle and Xiangxi cattle, indicating insufficient genetic variation to justify their classification as separate breeds. Collectively, these genomic data support the unified classification of Enshi cattle, Sinan cattle and Xiangxi cattle as a single breed, Wuling cattle.

Wuling cattle inhabit mountainous regions with large diurnal temperature fluctuations, heterogeneous vegetation and complex terrain and are an important source of draft animals in the area. This study identified several genes associated with skeletal and muscular development that have undergone positive selection, such as *EXT1*, *RIMS2*, *WDTC1* and *CDK19*. *EXT1* encodes a glycosyltransferase essential for heparan sulfate biosynthesis and skeletal development. Mice with *EXT1* mutations exhibit severe limb skeletal defects [24]. *RIMS2* encodes a presynaptic protein, regulates synaptic vesicle exocytosis, and plays a role in bone remodeling. Studies have revealed a significant association between *RIMS2* and bone weight in cattle [25]. *WDTC1*, an evolutionarily conserved inhibitor of lipid accumulation, regulates the transcription of lipid-related genes [27] and has been linked to milk composition in cattle [44]. *CDK19*, a member of the cyclin-dependent kinase (CDK) family, is homologous to *CDK8*. Previous studies have shown that *CDK8* expression is altered during bovine adipocyte differentiation [45], suggesting that *CDK19* may play a similar role in fat deposition. These results suggest that the climbing ability of Wuling cattle has been improved through long-term practice.

As a breed of cattle native to southern China, Wuling cattle must cope with a hot, humid, and complex climate, and the practice of grazing further intensifies the selective pressure on robustness. We identified a set of closely related genes under selection, such as *SLC9A1*, *SYNJ1* and *CYP19A1*. *SLC9A1* is highly conserved across mammalian species and plays a role in ion transport, acid–base balance, adaptation to high-energy diets and feed efficiency [23,46]. *SYNJ1* encodes a synaptic protein, and mutations in *SYNJ1* may reduce synaptic protein expression, which may limit the spread of pathogens such as Salmonella and contribute to immune competence in cattle populations [29]. *CYP19A1*, encoding aromatase, is crucial for estrogen biosynthesis. Mutations in *CYP19A1* affect bovine oocyte maturation, embryonic development, pregnancy success and placental development [47,48]. Further analysis shows that there are small but distinct selective signals among Wuling cattle populations from different regions; these signals are associated with environmental adaptations such as heat stress and vision. This suggests that different geographic environments may exert varying selective pressures on Wuling cattle, a factor that should be considered in future efforts to develop the genetic resources of this breed.

## 5. Conclusions

By analyzing whole-genome sequencing data from Wuling cattle, we show that Enshi, Sinan, and Xiangxi cattle share highly similar genetic compositions with minimal differentiation, supporting their classification as a single breed. Selective sweep analyses identified candidate genes involved in reproduction, growth, lipid metabolism and the immune response, including missense SNPs in *SYTL1* and *TAC4* that may underlie key adaptive traits. Group-specific analyses further revealed distinct selective signatures among the three populations, reflecting the influence of different geographical environments. The distinct selective signatures specific to each population suggest that local environmental factors have driven subtle adaptive differentiation at certain loci. These findings provide genomic insights for the conservation and utilization of Wuling cattle.

## Data Availability

The newly generated sequences for 20 Sinan cattle were deposited in the National Genomics Data Center (NGDC) under accession number PRJCA057460. The publicly available sequences were downloaded from the Sequence Read Archive (SRA) with the following project accession numbers: PRJNA176557 (Angus and Hereford cattle); PRJNA658727 (Weizhou cattle); PRJNA210523 (Hanwoo cattle); PRJNA658727 (Thawalam cattle); PRJNA762638 (Enshi cattle); and PRJNA1240382 (Xiangxi cattle).

## Supplementary Materials

The following supporting information can be downloaded at https://www.mdpi.com/article/10.3390/genes17091141/s1, Supplementary Material File S1 is available as Supplementary Material File S1.xlsx. Summary of detailed in-formation of the analysis. Table S1. Summary of sequencing data. Table S2. Distribution of SNPs in different regions of the genome annotated by ANNOVAR. Table S3. Genomic regions identified in Wuling cattle by iHS and CLR. Table S4 Nonsynonymous mutations in the selected region of Wuling cattle. Table S5 Selective regions within the Wuling cattle with a high proportion of indicine lineage. Table S6 Genomic regions identified in Enshi cattle by iHS and CLR. Table S7 Genomic regions identified in Sinan cattle by iHS and CLR. Table S8 Genomic regions identified in Xiangxi cattle by iHS and CLR. Table S9 Selective regions specific to Enshi cattle identified by iHS, CLR and XP-EHH. Table S10 Selective regions specific to Xiangxi cattle identified by iHS, CLR and XP-EHH.

## Abbreviations

The following abbreviations are used in this manuscript:

EUT	European taurine
EAT	East Asian taurine
EAI	East Asian indicine
SAI	South Asian indicine
*F_ST_*	Fixation index
WGS	Whole-genome Sequencing
SNP	Single-nucleotide polymorphism
LD	Linkage disequilibrium
PCA	Principal component analysis
NJ	Neighbor-joining
ROH	Runs of homozygosity
iHS	Integrated haplotype score
CLR	Composite likelihood ratio
XP-EHH	Cross-population extended haplotype homozygosity
CV	Cross-validation
